# A Skin-derived Antimicrobial Peptide Derived from Insulin-like Growth Factor-binding Protein 5 (AMP-IBP5) as Therapeutic Candidate for Psoriasis

**DOI:** 10.14789/jmj.JMJ22-0050-OT

**Published:** 2023-03-13

**Authors:** SAORI YOSHIBA, GE PENG, FRANÇOIS NIYONSABA

**Affiliations:** 1 Atopy (Allergy) Research Center, Tokyo, Japan; 1 Atopy (Allergy) Research Center, Tokyo, Japan; 2 Department of Dermatology and Allergology, Juntendo University Graduate School of Medicine, Tokyo, Japan; 2 Department of Dermatology and Allergology, Juntendo University Graduate School of Medicine, Tokyo, Japan; 3 Faculty of International Liberal Arts, Juntendo University, Tokyo, Japan; 3 Faculty of International Liberal Arts, Juntendo University, Tokyo, Japan

**Keywords:** psoriasis, antimicrobial peptide, AMP-IBP5, therapy

Antimicrobial peptide derived from insulin-like growth factor-binding protein 5 (AMP-IBP5) not only displays antimicrobial activities against invading pathogens but also has a wide range of immunomodulatory properties. In contrast to various antimicrobial peptides (AMPs) such as human β- defensins, cathelicidin LL-37 and S100A7 that are upregulated in psoriasis, insulin-like growth factor-binging protein 5, the parent protein of AMP-IBP5, is downregulated in psoriatic skin tissues. Psoriasis is an inflammatory skin disease characterized by epidermal hyperplasia, erythematous plaques, abnormal epidermal differentiation, and neutrophil infiltration into the epidermis. Although several AMPs have been implicated in the pathogenesis of psoriasis, the role of AMP-IBP5 remains unknown. Therefore, we aimed to investigate the effects of AMP-IBP5 in the pathogenesis of psoriasis using imiquimod-induced psoriasis-like mouse model and examined the underlying molecular mechanism.

Following subcutaneous administration of AMP- IBP5 into the psoriatic mice, we observed a remarkable reduction of dry scales and plaques, epidermal thickness, hyperkeratosis, parakeratosis, hyperplasia of dermal vessels and neutrophil infiltration in psoriatic mice compared to normal mice. Consistently, AMP-IBP5 administration markedly suppressed the expression of keratinocyte differentiation markers, including involucrin and loricrin, inflammatory cytokine tumor necrosis factor-α, antimicrobial peptides (cathelicidin and S100A proteins), and various angiogenesis factors from the psoriatic lesional skin. To clarify the molecular mechanism by which AMP-IBP5 alleviates psoriasis symptoms, we focused on low-density lipoprotein receptor- related protein 1 (LRP1), which regulates inflammatory responses and keratinocyte differentiation. The expression of LRP1 was noticeably decreased in the lesional skin of patients with psoriasis compared to that nonlesional or normal skin tissues, suggesting that dysregulation of LRP1 in the epidermis may be involved in the pathogenesis of psoriasis. Interestingly, administration of LRP1 antagonist, receptor-associated protein (RAP), exacerbated psoriasis and abolished AMP-IBP5- mediated improvement in psoriasis, suggesting that LRP1 is crucial in the pathogenesis of psoriasis and that LRP1 pathway is necessary for AMP- IBP5-induced improvement in psoriasis. Collectively, we provide evidence that AMP-IBP5 might be a novel potential therapeutic target for the treatment of psoriasis.

## Funding

This work was partially supported by a Grant-in-Aid for Scientific Research from the Ministry of Education, Culture, Sports, Science and Technology of Japan to FN (grant number: 26461703 and 21K08309), a grant from the National Eczema Association (grant number: NEA21-ERG159 and NEA22-ERG169) and Vichy Exposome Grant to GP.

## Author contributions

Conceptualization: SY, GP and FN; Data curation: SY and GP; Funding acquisition: GP and FN; Methodology: SY, GP and FN; Supervision: GP and FN; Visualization: SY and GP; Writing, original draft preparation: SY and GP; Writing, review and editing: SY, GP and FN. All authors read and approved the final manuscript.

## Conflicts of interest statement

The authors have no conflicts of interest to report.

